# Exploration of Nurses’ Knowledge, Attitudes, and Perceived Barriers towards Medication Error Reporting in a Tertiary Health Care Facility: A Qualitative Approach

**DOI:** 10.3390/pharmacy6040120

**Published:** 2018-11-05

**Authors:** Eman Ali Dyab, Ramadan Mohamed Elkalmi, Siti Halimah Bux, Shazia Qasim Jamshed

**Affiliations:** 1 Department of Pharmaceutics, Faculty of Pharmacy, Tripoli University, Tripoli 42300, Libya; eman_diab2008@yahoo.com; 2 Department of Pharmacy Practice, Faculty of Pharmacy, Universiti Teknologi Mara, Puncak Alam 42300, Malaysia; edriph@gmail.com; 3 Department of Pharmacy Practice, Kulliyyah of Pharmacy, International Islamic University Malaysia, Kuantan 25200, Malaysia; sitihalimah@iium.edu.my

**Keywords:** medication error reporting, nurses’ attitudes, qualitative study, barriers, medication error

## Abstract

Medication error reporting (MER) is an effective way used to identify the causes of Medication Errors (MEs) and to prevent repeating them in future. The underreporting of MEs is a challenge generally in all MER systems. The current research aimed to explore nurses’ knowledge on MER by determining their attitudes towards reporting and studying the implicated barriers and facilitators. A total of 23 nurses were interviewed using a semi-structured interview guide. The saturation point was attained after 21 interviews. All the interviews were tape-recorded and transcribed verbatim, and analysed using inductive thematic analysis. Four major themes and 17 sub-themes were identified. Almost all the interviewees were aware about the existence of the MER system. They showed a positive attitude towards MER. The main barriers for MER were the impacts of time and workload, fear of investigation, impacts on the job, and negative reactions from the person in charge. The nurses were knowledgeable about MER but there was uncertainty towards reporting harmless MEs, thus indicating the need for an educational program to highlight the benefits of near-miss reporting. To improve participation strategies, a blameless reporting culture, reporting anonymously, and a simplified MER process should be considered.

## 1. Introduction

The main principle of giving medication to the patient is to serve them in order to restore his/her health without any harm [1]. Adverse events and medical errors are the main issues threatening the patient’s safety, and are awkward predicaments in nearly all healthcare systems [2]. The World Health Organization (WHO estimated that millions of people suffer injuries directly attributed to medical care, and many are preventable [3], although prevalence in developing countries is reported to be higher than developed nations [4]. In Southeast Asian countries, the reported administration error rates ranged from 15.2% to 88.6% [5]. In Taiwan, a study mentioned that the overall rate of the medical incidents ranged from 30% to 47.6%, and most of them were related to MEs [6]. In a Malaysian study, Johari et al. reported 2572 reported cases of ME in 2009 [7]. The prevalence of ME among geriatric patients was 25.17% [8] whereas in paediatrics this figure was 11.7% [9]. A recently published four-year retrospective study reported that the total number of paper-based ME reports submitted to the National Medication Error Reporting system (NMER) was 17,357, but only 0.3% of MEs were in the administration stage [10]. The percentage in the administration stage is very low, representing paper-based reports only (excluding online submitted MEs reports) collected by the NMER system, as well as the effect of underreporting. The annual estimated cost of MEs in Malaysia was estimated to be 111,924 Malaysian Ringgit (MYR) [10]. Clinically, MEs can have small to severe consequences for patients. It was reported that the total number of MEs in the administering stage was 166, where 1% had fatal consequences, 20% were serious, 32% were significant, and 46% were nonsignificant [11].

The identification of trends and patterns of MEs were the main reasons for establishing a ME reporting system [12]. The effectiveness of all these systems depend on their ability to document the occurred MEs. Establishing guidelines for medical error reporting (MER) is not enough, as the healthcare practitioners (reporters) play a vital role in the MER process. The healthcare practitioners’ knowledge about MER, their attitudes toward reporting MEs, and perceived barriers toward MER are important factors which determined the success of MER systems. Health care professionals in general and nurses in particular are responsible for MER. It has been reported that the MEs are underreported in all countries. Nurses’ knowledge and attitudes as well as barriers and facilitators toward MER among nurses in Malaysia are little-studied issues and warrant investigation.

This study is intended to attain deeper insight into the knowledge and attitudes held by Malaysian nurse practitioners towards the ME concept and MER process, as well as to investigate the barriers which prevent nurses from reporting their MEs.

1. What do nurses know about the ME and MER system?
2. What are the nurses’ attitudes toward MER?
3. What are the barriers which could hinder nurses from reporting their MEs?
4. What are the factors which could facilitate MER among nurses?

## 2. Methods

### 2.1. Study Design and Setting

The study was conducted after getting approval from both the Medical Research Ethics Committee Ministry of Health Malaysia (NMRR-15-2485-24709) and the International Islamic University of Malaysia Research Ethics Committee (IREC 446). Use of a qualitative method (in-depth interviews) provides flexibility and efficiency in collecting data related to personal feelings, attitudes, and experiences [13] and hence, these methods are used for the exploration of the participants’ experiences towards medication error reporting. The individual interview method was chosen over focus group discussions because of privacy, suitability, and comfort zone issues for shy and hesitant participants [14]. This gives liberty to the participants to elaborate his/her answers without distractions from others [14]. The current research was executed in a tertiary healthcare facility of Kuantan city, Pahang, Malaysia.

### 2.2. Participants

The target study population was nurses practicing in different units of the hospital. No specific inclusion and exclusion criteria were applied, and thus all nurses were eligible to participate in the current research. The participants were recruited by using convenience and snowball sampling technique. The snowball method helps to recruit hidden subjects which cannot be easily found [15]. The first participant was a nurse from the medical unit and was given information on the details of the research along with an assurance of confidentiality and anonymity. After her acceptance she was asked to fill in demographics form and sign the consent form. The interviews were recorded by using Audio Recording Titanium Software^®^ version 8.5.5 (AATSystems, Kent, UK), and notes were taken during the interviews. After finishing the first interview, the interviewee was asked to nominate the next participant. Thus, she made a referral to the next nurse, with the same pattern for the subsequent interviews. Thus, a chain referral technique followed throughout the research study. The number of participants was determined once the saturation point was achieved. The saturation point occurs when no new concepts and themes emerge [14,16,17]. Two extra interviews were conducted to confirm the saturation point. The field supervisor also helped in recruitment process. Participation was simply on a voluntary basis, and they were informed that anytime during the course of research they could withdraw. Moreover, they were assured that their confidentiality and anonymity would be maintained. Only the research team had access to records. The purpose of the study was explained to each participant before the interviews commenced and therefore, all the participants were asked to sign an “informed consent form” followed by the addition of their demographic characteristics. A total of 23 nurses were interviewed.

### 2.3. Procedure and Interview Process

In-depth interview sessions were conducted using a semi-structured interview guide in June 2015. This guide was developed on the basis of prior published studies related to MER among health care professionals [18,19,20]. The purpose of using this guide was to make sure that all important issues about the topic were covered in the interviews [13,21]. The medium of communication during the interview was primarily English, followed by a couple of interviews being conducted in Bahasa Malayu i.e., the native language of participants. The field supervisor agreed to work as a research assistant and a translator and assisted the nurses who could not understand the English language. As a result, participants who expressed their thoughts in the Bahasa Malayu language were also included. Each interview lasted for about 30–45 min. Venue was chosen as per proximity to nurses’ working units, their preference of level of comfort/privacy, and the level of noisiness. Places such as the library private room, the seminar room, and nurses’ rooms were selected as the venue for interviews. The discussion was focused on several major issues; the nurses’ knowledge, experiences, and perceptions about ME and MER, exploring their attitudes towards MER, comprehending the factors which might prevent the nurses from reporting their MEs, and those factors which would promote MER among nurses. Probing questions were asked to provoke more details from interviewees [13,21].

### 2.4. Data Analysis

**Figure 1:**
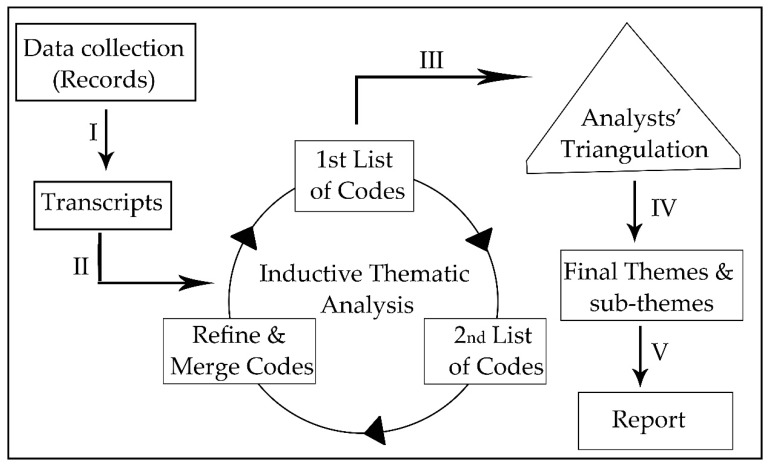
The study process flowchart. (I) transcription step, (II) data analysis step, (III) analysts’ triangulation method (two researchers performed analysis and third person resolved any disagreement), and (IV) final result.

The data analysis was performed using the inductive thematic analysis approach. The participants’ approved transcripts (transcribed verbatim) were coded as (N1, N2, …, N23). The process is illustrated in Figure 1. The analysis followed a cyclic pattern, where it started by familiarization stage, generation of initial codes stage and revision stage to refine the emerged codes [22,23]. The transcripts were analysed again by another researcher to validate the resulted themes [22], and a third person’s opinion was sought to resolve any disagreement between the previous analyses [24,25].

The current research followed an established criterion for maintaining quality in qualitative research and thus follow the standards of Guba and Lincoln [26] for generating credibility, transferability, dependability, and confirmability. For strengthening the credibility of the research there was a continuous interaction with the participants, with checking of interpretations against interview transcripts. A review with the participants was undertaken. Contrary to quantitative research, the aim of qualitative is not generalizability but to observe and execute transferability. Therefore, a detailed description of the participants’ experiences helped the researchers identify the patterns of social relationships in reporting MEs and as well as the cultural backgrounds of the participants who reported hesitancy in reporting. For dependability purposes, external audit criteria were put in place, and a researcher not involved directly in research helped in the evaluation of interpretation and conclusions with respect to the data collected. For establishing confirmability, not only a conformability audit (as mentioned above) but also triangulation and reflexivity were maintained. In terms of establishing triangulation, both methodological triangulation (i.e., the research followed the quantitative design after qualitative inquiry) and analyst triangulation (i.e., using different analysts to review the findings) were maintained, whereas for reflexivity, a reflexive note-sheet was used to record the methodological parameters and logistics involved. Interviewees were female (*n* = 22); of Malay race (*n* = 22); holding a diploma (*n* = 21). The nursing diploma is a 3-year course in Malaysia and on its successful completion one can register with Nursing Board Malaysia and work as a staff nurse. All participants were full time employees. Half of the participants (*n* = 12) have working experience of more than 11 years. Slightly more than three-quarters of the participants (*n* = 18) had not reported any MEs over the prior 12 months. The interviewees were attached to different units in the hospital such as intensive care units (ICUs), medical units, critical cardiac units (CCUs), accident and emergency unit (A&Es), orthopaedic units, neonatal intensive care units (NICUs), and paediatric units. The demographic characteristics of participants are presented in Table 1.

## 3. Results

**Figure 2:**
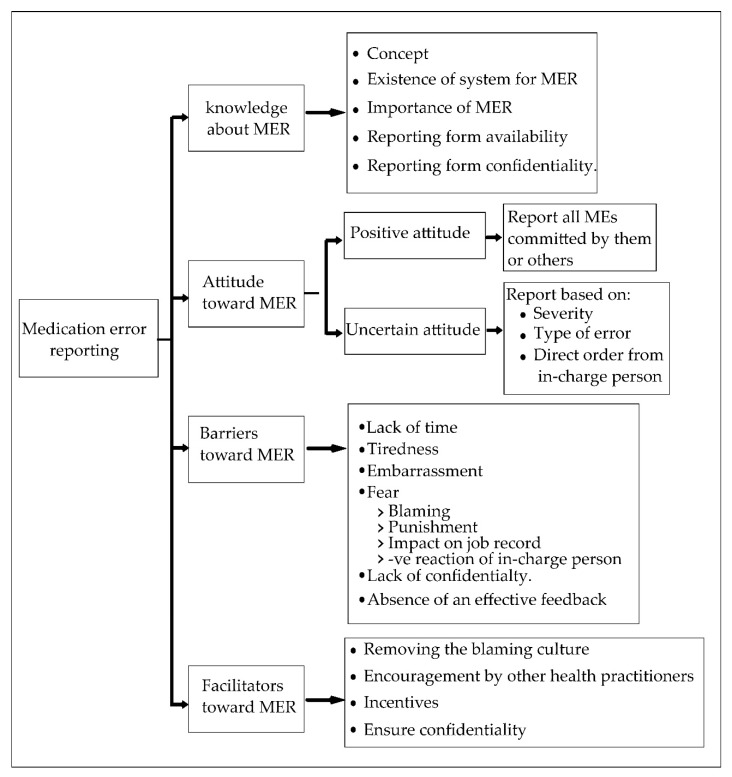
Emergent themes and sub-themes. MER: medical error reporting.

Four major themes and 17 sub-themes were emerged: knowledge about MER, attitudes toward MER, barriers toward MER, and facilitators to improve MER process. Figure 2 represents the emerged themes and categories.

### 3.1. Knowledge about MER

#### 3.1.1. Concept of ME

The nurses were asked about their understanding of the ME concept. Almost all of them correctly understood the concept of ME. Moreover, they linked its meaning to five/seven rights, while others just gave simple and general answers like “giving incorrect medication to the patient”.

“Medication error is an error when giving medication including dosage and also the type of medication, make sure to follow the 7Rs practice in the hospital.” (N1)

“Medication error is when something unwanted occurs such as wrong medication is given to the patient.” (N7)

“Medication error means giving wrong medication to the patient, which includes wrong dose, wrong route, and wrong documentation.” (N13)

#### 3.1.2. The Existence of a System for MER and the Importance of MER

All the interviewees were aware about the existence of MER system and the importance of MER. They stated that data collected by MER can be used as an indication of the quality of health service provided to the patient. It can be used to improve this service by carrying out root-cause analysis for the MEs reports, and the reported data can be utilized for learning purposes. In other words, ME reports can be used as good resources to help nurses in avoiding repeating the same errors again in future.

“Yes, we have a system for medication error reporting […] And, it is very important because it involves the quality of service which is being given to the patient and it is very important to monitor ME.” (N1)

“It is important because we want to improve the way of delivering care and serving the patient. To learn from reports, where and which thing can be done. So we have more information about what has been done and their consequences.” (N3)

“It is important because we want to detect what is ME and to prevent it from happening again.” (N7)

“Normally, we do root-cause analysis to find out when and how this happened. Sometimes it comes from the wrong prescription like wrong dose or wrong route or wrong frequency and then we find out how that happen and try to tackle.” (N5)

“It is to guide our practice […] Not add more error to this collection […] To avoid ME in future […] It is considered as a good resource.” (N4)

#### 3.1.3. The Availability and Confidentiality of the Reporting Form

The majority of interviewed nurses claimed that during their practice, they did not report MEs, since until the time of interviews they did not commit any error. As a result, most of them did not see the reporting form and some of them had seen it but they did not remember its content.

“The reporting form is available in the pharmacy department.” (N5)

“I have not seen the reporting form before. Because, so far, I did not make any error.” (N8)

The nurses were asked about their opinions on the reporting form.

“I have seen it; it is easy to fill, it does not need modification or re-designation.” (N1)

“The report is not too detailed like describing everything, but it underlines or highlights when the medication was given to the patient.” (N3)

### 3.2. Attitude of Nurses toward ME Reporting

The nurses were asked about their attitudes toward ME reporting. The majority of the participants had a positive attitude toward reporting of MEs, whether these MEs caused a serious side effect to the patient or not. The other group had uncertain attitudes and they tended to report the MEs which led to harm to the patient only.

#### 3.2.1. Positive Attitude

The majority of nurses claimed that they report all encountered MEs immediately. They reported them irrespective to their seriousness or the level of patient’s harm due to the error.

“Nothing affects my decision to report, once the error occurs it should be reported.” (N4)

“It is not a matter of choice.” (N7)

“Once I detect an error, I cannot just ignore it, and I straightforward report it [...] We must make a report also because this is ME, and we must report whether it is serious or not.” (N2)

“Here in A and E department, it does not matter if the error is big, mild, or small, it must be reported.” (N8)

#### 3.2.2. Uncertain Attitude toward ME Reporting

During the discussion with the participants about their attitude toward MER, some participants showed uncertain attitudes towards MER. They would report MEs only based on another factor such as the severity of the ME or route of administration, or when they received a direct request form the person in-charge.

“If the error caused big and serious complication I have to report.” (N17)

“Based on the patient, I will see the effect on the patient first. My first concern is the patient, I will not report unless something happens to the patient. In this case, the doctor gives antidote and then there is an investigation and eventually, they will revert to me.” (N9)

One nurse related the medication error reporting to the dosage form of administered medicine. He believes that errors are serious when the medication given by the intravenous route, and this type of error should be reported, while those resulting from oral or topical administration should not be reported.

“Based on the route of administration IV it should be reported.” (N18)

One nurse insisted that she reports only if the person in charge requests her to fill the reporting form.

“I just inform the sister and the doctor, and let them choose to fill the form or not but as for investigation, I will come and join them” (N13)

Before reporting, the nurses think of the problems that will be faced after reporting their errors. This has a high effect on their decision to report or not.

“Some nurses, at first, they think about what happen and the problems associated with reporting, so they do not report.” (N12)

#### 3.2.3. Reporting of Others’ Errors

Some of the interviewees stated that they do not have any problems in reporting MEs committed by other staff. They believe that the reporting of MEs is better for both the nurses and also for the patient, whether the MEs have been committed by themselves or by other health care professionals. While the other group insisted that everyone is responsible for reporting his/her own initiated MEs.

“I will report if other staff nurse made a mistake.” (N1)

“I will report errors committed by others because this is in the best interest of the patient, and also it would help things go smooth in the future, for example, patient allergy …” (N3)

“If I made a mistake I would inform, also if others from my colleagues made a mistake, I would still inform.” (N6)

“No, I report only my errors. If my colleagues made mistakes, I would just advise her to report, but I will not report her error.” (N8)

### 3.3. Barriers towards Medication Error Reporting

There are many barriers towards MER which were mentioned by the interviewees. These barriers are heavy workload, lack of time, tiredness, embarrassment due to reactions of peers and family, and fear of disciplinary action.

#### 3.3.1. Lack of Time

The main barrier for MER mentioned by the interviewees was time. They considered that the ME reporting is a time consuming process. As described by the participants, the problem is not the time needed to fill the MER form. The problem appears after filling the MER form when the investigation takes place in order to discuss the causes which led to the ME.

“We will be exposed to so many questions […] long time […] time to discuss the ME that was reported […] investigations take time. No other problems, just that it takes time to report and then questions from pharmacist or doctors. We do not have time for reporting. It is a long story and takes much time.” (N4)

“Sometimes, I decide not to report. Because, if there is an investigation we have to be presented, as you know it will take a long time and we will be all inconvenient.” (N9)

#### 3.3.2. Tiredness

The nurses are responsible for inpatient care, this responsibility requires them to accomplish many physical activities. Performing these activities make nurses tired, when they are exhausted, a low number of ME reports will be received from them.

“Sometimes, we are tired. Once we are tired we decide not to report.” (N4)

#### 3.3.3. Embarrassment

The post-embarrassment feeling has been pinpointed by interviewees as another barrier toward MER. As a result, they tend to hide their MEs and never report them.

“Facing the embarrassment from my family and friends is tough. They will blame us.” (N4)

“They (family and friends) understand because these are not things that a person does on purpose. But facing them still difficult.” (N9)

#### 3.3.4. Fear

Fear from the legal problems has been addressed by the interviewees as a barrier towards MER.

“I fear from legal problems and disciplinary actions from the hospital.” (N8)

“Sometimes, I do not want to get into issues, I do not want people to come to ask me for investigation later.” (N2)

The effect of reporting on the personal job record is another factor which might prevent nurses from reporting.

“If I report this will affect my record because everything will be recorded in my personal record.” (N9)

“Fearing others, especially the investigation, because in Malaysia all errors must be reported to your job record and they do disciplinary action.” (N4)

#### 3.3.5. Negative Reaction from Sister In-Charge

The response of managers toward nurses who report their MEs were important factors which prevented nurses from reporting their MEs. It has been noticed that most of the interviewees insisted that receiving a negative reaction from senior nurses is a normal response when the MEs occur. As they always tend to blame and scold the nurses if they commit MEs. They believe that guidance is the main role of senior nurses during their practices, not blaming the nurses.

“The sister will monitor me more.” (N8)

“Negative reaction from sister and matron […] they must not punish the staff, they must guide the staff and follow the staff and ensure that the stuff follows the standards.” (N4)

#### 3.3.6. The Confidentiality of the Reporting Form

The reporting form which is used to report ME is a strict confidential form. Filling this type of the reporting requires the reporter’s details such as name, signature and contact details. Some nurses did not report their MEs because of this issue and they wish if they can report by using an anonymous reporting form. Consequently, the number of reports will increase by use of anonymous MER forms.

“I prefer to fill anonymous form […] Because I feel shy and would not work further. Also, I would feel sorry for the patient. So, I prefer to fill the form without names.” (N2)

“I prefer to fill the anonymous form as it is good for us. If mistakes have been done, the news of medication errors should be displayed without names being mentioned. In the future, if the people know that this person made a mistake, people would decide not to deal with this person again. This will damage the confidence of the nurse. In the future, they will not report and there will be no chance to learn from the mistakes.” (N6)

“Off course, if no names mentioned the number of reports will increase.” (N8)

#### 3.3.7. Absence of Effective Feedback

The lack of feedback from the hospital managers:

“No one goes through all the errors and give me a feedback.” (N7)

“I did not receive any feedback for my ME report.” (19)

### 3.4. Facilitators to Improve ME Reporting

The nurses were asked about the factors which could encourage them towards MER. Their main concern revolved around removing the blaming culture. They believed that if no one scolded them about their errors they would definitely report their errors.

“Remove the blaming culture. The matron and sister in charge should guide the staff not blame them.” (N4)

“Tell the matron that if any person is involved in a medication error, she shall not be scolded.” (N7)

Another factor which was addressed by the interviewees was getting encouragement from others toward MER. Regarding this, there were two different opinions: first, some nurses insisted that they did not need any encouragement from others because they thought the MER is an integral part of their responsibility; on the contrary, other nurses welcomed encouragement by other health practitioners such as a doctor, matron, or even their colleagues.

“There is no need to encourage us because this is our duty.” (N12)

“The sister in charge encouraged me to report.” (N9)

“Actually, among us, we as nurses encourage each other to report errors; also the sister in charge encourages us to do that.” (N8)

Few nurses highlighted the incentives as an effective way to encourage nurses to be more meticulous to report MEs.

“Giving monetary rewards to the nurses.” (N3)

The confidentiality of the reporting form is an important factor, some of them preferred to fill anonymously to avoid the embarrassment and being reprimanded by the authorities.

“I prefer to fill the form with no names and it is better not to include names.” (N2)

“I think as long as they can ensure the confidentiality of the person who reported, we will feel safe.” (N9)

## 4. Discussion

This is an exploratory study intended to investigate the knowledge and attitude of nurses towards MER. The current research is also anticipated to address the barriers and facilitators towards MER among nurses, attached to different medical wards in the hospital.

The interviewed nurses reflected on the basic knowledge of concept of ME and MER. They reported awareness about the presence of ME reporting system, guidelines, and the importance of the MER. This might be attributed to the frequent talk sessions and training courses such as the continuing nursing education program (CNE), in addition to the encouragement from the nurse leaders (head nurses, supervisors and directors). This finding is consistent with the previous studies conducted in Malaysia [7,27]. Wei and his colleagues reported that the Malaysian nurses had baseline knowledge regarding MEs, whereas Johari et al. reported that Malaysian nurses had good knowledge level regarding medication administration safety. However, most of the interviewees were not familiar with the content of the ME reporting form due to their lack of contact. The low involvement of nurses toward MER was not related to the lack of knowledge about the MER or due to the lack of information about the process of incidents reporting, as reported in previous studies [28,29]. Handler and his colleagues. reported that the lack of information on how to report ME among nurses as a barrier for MER and this needs an immediate action and should be on higher priority towards improving MER among nurses.

The willingness of nurses to report MEs has great impact on MER practices. Respondents had two contradictory attitudes toward MER. Positive attitudes towards reporting all MEs are found to be in accordance with what has been stated in Malaysian medication error guidelines [30], while an uncertain attitude was also stated where participants were keen to report major errors only. In this case, the minor errors and near-miss errors most likely will not be reported, in line with the previous studies [31,32,33,34]. Martowirono et al. reported that the MEs with minor consequences were lesser reported. Reporting of near-miss errors gives valuable lessons without harming the patient [35]. In such situations, a seminar discussion with the experienced nurse managers about benefits of near-miss error reporting can be a useful tool to improve near-miss reporting rate among nurses.

The current research revealed that most nurses have positive attitudes toward ME reporting. However, factors such as lack of time for reporting, lack of reporting culture without being blamed, lack of effective feedback, and fear are considered as main reasons for underreporting problems among the participants. These findings were consistent with the study conducted in Taiwan [36] where fear was cited as the fundamental projecting factor in underreporting.

Despite the positive attitude of nurses towards the MER, they revealed that they did not report MEs due to barriers like paucity of time, already in accordance with the studies done in Taiwan and Canada [6,37]. Lack of time could be a reflection of heavy workload, as in many instances a limited number of nurses take care of many patients. On the other hand, lack of reporting can be related to the MER process, which starts informally by informing the doctor, pharmacist, and the nurse director, and gradually follows by formally filling the MER form. Increasing the number of staff and simplifying the MER process are two recommended remedies to overcome time constraint issue. There is an evidence that decreasing number of patients per nurse lowered the mortality rate and increased the cost. However the four patients per nurse ratio is still considered as cost-effective [38].

Fear is mentioned by the interviewees as another barrier toward MER. The interviewees claimed that they do not fear losing their job, but they fear from the patient’s reactions if the ME was revealed to them. Also, they fear from the nurse managers and matron’s reaction against the reporters as they will be blamed for their MEs. This can have a negative impact on the number of reported errors. Such phenomena are consistent with previous studies [6,32] as Lin and Ma reported that around two-third of the participants were worried about getting punished, taking the medical responsibility, and loss of patient’s trust. Strategies like providing a blame-free reporting culture, as well as using anonymous reporting forms could be intensely helpful to diminish the “fear feeling” among the nurses.

As emphasized in the current research the confidentiality of the reporting form is an important matter as many prefer to fill out the anonymous form, escaping embarrassment and liabilities from other health care professionals. The effect of using anonymous reporting forms has been addressed in previous studies [35,36,39,40]. Suresh et al. highlighted that a wide range of MEs were reported by using anonymous online reporting form.

The concept of under-reporting and barriers involved in reporting is not only related to developing and transitional economies. A recently published study from a community hospital California reported that error reporting was laborious in terms of time management, and was related to fear of liability, lawsuits, or disciplinary action [41]. 

The findings of this study will be helpful for policy makers to refine the applied strategies which used to improve nurses’ participation in the MER process, leading to increased patient safety inside healthcare facilities. Moreover, a training and educational program regarding patient safety and near-miss reporting should be provided for the nurses to highlight the importance of near-miss reporting and to maintain safe medication administration without harming the patients. For the future, a thorough study which includes other health care practitioners such as doctors, nurses, and pharmacists could be done; this would divulge perspectives in diverse stages.

## 5. Conclusions

The findings of this study reflected the familiarity of nurses with the MER system. The attitudes of nurses had a great impact on MER, especially their attitudes toward reporting near-miss errors and harmless MEs. Reasons for underreporting include lack of time, workload, embarrassment, fear of investigation, types of reporting forms, and absence of feedback on the previously collected ME reports. For improving the involvement of nurse in MER, confidentiality of the reporters coupled with a blame-free culture are identified strategies that might support improved involvement of nurse in MER.

## 6. Limitations

The findings are related to interviewed nurses only and may not be generalizable to other tertiary settings, although the regulations which regulate the nursing jobs in Malaysia are same in all Malaysian states. This would mean that there is a possibility that the knowledge and attitudes of nurses in another Malaysian hospital might be like those of the current research participants. The study being of exploratory qualitative type does not warrant generalizability itself. A selection bias cannot be ruled out, as the first interviewee was selected by the convenience method. Moreover, a chance of loss of meaning due to the translation strategy cannot be put aside. We tried to overcome this issue, and in fact, the chosen translator was one who comprehended the culture and field of interviewees well. In this way we attempted to minimize the risk of misinterpretation or inaccurate translation, as the translator was familiar with the terms and ways of expression used by the interviewees [42].

## Figures and Tables

**Table 1:** Interviewees socio-demographic characteristics (*n* = 23).

Characteristic		Number (*n* = 23)	Percentage (%)
Gender	Female	22	95.7
	Male	1	4.3
Race	Malay	22	95.7
	Chines	1	4.3
Age	≤30	6	26.1
	30–40	14	60.9
	41–50	2	8.7
	51≥	1	4.3
Education level	Diploma	21	91.3
	Bachelor	2	8.7
Experience in years	≤5	5	21.7
	6–10	6	26.1
	≥11	12	52.2
Practice site	Medical unit	4	17.4
	ICU ^a^	9	39.1
	CCU ^b^	2	8.7
	A & E ^c^	3	13
	Orthopaedic unit	2	8.7
	NICU ^d^	1	4.3
	Paediatric unit	2	8.7
Number of reports in the last 12 months	Never report	18	78.3
	≥1	5	21.7

(^a^) Intensive care unit. (^b^) Critical cardiac unit. (^c^) Accident and emergency unit. (^d^) Neonatal intensive care unit.
